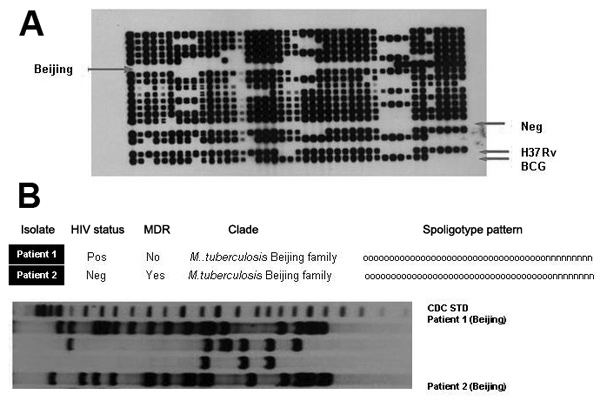# *Mycobacterium tuberculosis* Beijing Strain, Bamako, Mali

**DOI:** 10.3201/eid1602.090501

**Published:** 2010-02

**Authors:** Bassirou Diarra, Sophia Siddiqui, Dramane Sogoba, Brehima Traore, Mamoudou Maiga, Janice Washington, Anatole Tounkara, Michael A. Polis

**Affiliations:** Project SEREFO-NIAID/University of Bamako Research Collaboration on HIV/TB, Bamako, Mali (B. Diarra, D. Sogoba, B. Traore, M. Maiga, A. Tounkara); National Institute of Allergy and Infectious Diseases, Bethesda, Maryland, USA (S. Siddiqui, M.A. Polis); US Food and Drug Administration, Silver Spring, Maryland, USA (J. Washington); 1 These authors contributed equally to this article.

**Keywords:** Tuberculosis and other mycobacteria, pulmonary tuberculosis, Mycobacterium tuberculosis, Beijing strain, Mali, spoligotyping, antimicrobial resistance, bacteria, drug susceptibility pattern, letter

**To the Editor:** *Mycobacterium tuberculosis* has >36 identified genotype families (*1*). Four genotypes cause 35% of documented cases of active tuberculosis (TB): Beijing (10%–11%), Latin American–Mediterranean (9.3%), Haarlem (7.5%), and the X clade (7%) (*1*,*2*) The Beijing clade strains, reported in 1995 from the People’s Republic of China, are widely recognized as highly pathogenic with a possible predilection for multidrug resistance (*3*). Predominant in Asia, these strains have been documented in other parts of the world (*1*,*4*,*5*). The virulence, propensity to become resistant, and distinct geographic distribution of the Beijing clade suggest it may have some adaptive advantage in producing disease in humans. Limited data suggest that its presence in Africa is low (*2**,**4**,**5***)**.

In Bamako, Mali, 2 patients with active pulmonary TB came to the research clinic at Point G Hospital, affiliated with the University of Bamako Medical School, for recruitment under a US National Institute of Allergy and Infectious Diseases’ institutional review board–approved protocol. The first patient, a previously healthy 34-year-old man, sought treatment in March 2008. He had a 3-month history of fever, cough, shortness of breath, and left-sided chest pain; respiratory rate of 24/min; temperature of 36.8ºC; and pulse rate of 68/min. He weighed 60 kg. His leukocyte count was 8,700 cells/μL, and he was positive for HIV-1 with a CD4+ T-cell count of 468 cells/μL. He reported contact with persons from other countries in Africa, China, and other parts of Asia.

Chest radiograph showed a cavitary lesion on the left upper lobe and opacities throughout the left lung. Three sputum samples collected 3 days apart were digested and decontaminated with N-acetyl-L-cysteine, 4% NaOH; concentrated by high-speed centrifugation; stained with auramine-rhodamine; and evaluated by using fluorescent microscopy. The many acid-fast bacilli (AFB) seen were identified by using nucleic acid probes (AccuProbe, Gen-Probe, San Diego CA, USA). Antimycobacterial drug susceptibility was determined by using a manual indirect susceptibility test (mycobacterial growth indicator tube, AST SIRE System; BBL, Becton Dickinson, Franklin Lakes, NJ, USA) showed the isolate sensitive to isoniazid (0.1 μg/mL), rifampin (1.0 μg/mL), and ethambutol (3.5 μg/mL) but resistant to streptomycin (0.8 μg/mL). Spoligotyping using a commercially available kit (Spoligotyping Isogen Life Science, De Meern, the Netherlands) showed characteristics of the Beijing clade (Appendix Figure, panel A) (*6*).

The patient began treatment with the standard first-line regimen of isoniazid, rifampin, pyrazinamide, and ethambutol fixed-dose combination (Svizera Laboratory, Mumbai, India) according to Malian National Guidelines. Follow-up sputum samples at 13 and 18 weeks of treatment were smear- and culture-negative for AFB.

The second patient, a 28-year-old woman, sought treatment in July 2008. For 1 year, she had received first-line and retreatment regimens that failed to clear her sputum of AFB. She had begun second-line treatment for multidrug-resistant disease 2 days earlier. She had a history of fever, cough, and weight loss; temperature of 37.1ºC; heart rate of 104 beats/min; respiratory rate of 24/min; and blood pressure of 90/60 Hg mm. She weighed 49 kg. Leukocyte count was 9,400 cells/μL. Serologic results for HIV-1 and -2 were negative. Chest radiograph showed a right apical cavitary lesion and a fibrotic lesion in the right middle lung field. She did not recall any exposure to TB. She worked as an assistant at a local telephone center.

Two sputum samples, processed as described above, were positive for, and Gen-Probe testing confirmed, *M. tuberculosis*. According to antimycobacterial susceptibility testing, the strain was resistant to isoniazid (0.1 μg/mL), rifampin (1.0 μg/mL), ethambutol (3.5 μg/mL), and streptomycin (0.8 μg/mL). Spoligotyping confirmed the strain as Beijing clade, and restriction fragment length polymorphism (*7*) confirmed that it differed from that of patient 1 (Appendix Figure, panel B).

The relevance of different genotypes, such as the Beijing clade, to disease progression is being studied. Evidence indicates the genotype may factor in transmission or pathogenesis. In a study in Cape Town, South Africa, disease produced by the Beijing clade increased exponentially over time, suggesting a possible pathogenic advantage; although most cases were drug susceptible, the likelihood of unsuccessful treatment was greater than for non-Beijing variants (*8*). Although the Beijing clade does not appear to have greater propensity than non-Beijing genotypes for acquiring resistance, certain variants within the group that become multidrug resistant may be more likely to acquire such resistance. Beijing strains particularly may tend to acquire resistance more easily than others under conditions of suboptimal treatment (*9*). In Cape Town during 2000–2003, the Beijing clade as a cause of disease in children increased from 13% to 33%, suggesting a selective advantage in transmissibility and disease production (*10*).

These cases highlight the need to diagnose disease and resistance early and to begin appropriate treatment in TB-endemic countries. Knowledge of circulating strains and their resistance patterns is essential to developing effective programs to curtail the spread of TB within the country and the region; in this era of globalization, it is required for the successful control of TB worldwide.

## Supplementary Material

Appendix Figure A) Identification of isolates of the *Mycobacterium tuberculosis* Beijing clade (*6*) by using spoligotyping. The spoligotype pattern of the *M. tuberculosis* Beijing clade is characterized by the absence of hybridization of spacers 1–34 as shown, in combination with hybridization of spacers 35–43. Negative control (Neg.) shows absence of all spacers. For comparison, H37Rv (a laboratory strain) and *M. bovis* BCG show different patterns of spacers. Also shown are other clades not identified. B) Restriction fragment length polymorphism (*7*) patterns of strains from the 2 patients. Patterns confirm that both strains belong to the Beijing clade. However, patterns indicate different strains and confirm lack of direct transmission between the patients. The 2 additional clades shown (not from these patients) illustrate differences between the Beijing and other clades.